# Management of multicellular senescence and oxidative stress

**DOI:** 10.1111/jcmm.12074

**Published:** 2013-06-22

**Authors:** David D Haines, Bela Juhasz, Arpad Tosaki

**Affiliations:** a Department of Pharmacology Faculty of Pharmacy Health and Science Center, University of DebrecenDebrecen, Hungary

**Keywords:** apoptosis, necrosis, autophagy, necroptosis, senescence, oxidative stress

## Abstract

Progressively sophisticated understanding of cellular and molecular processes that contribute to age-related physical deterioration is being gained from ongoing research into cancer, chronic inflammatory syndromes and other serious disorders that increase with age. Particularly valuable insight has resulted from characterization of how senescent cells affect the tissues in which they form in ways that decrease an organism's overall viability. Increasingly, the underlying pathophysiology of ageing is recognized as a consequence of oxidative damage. This leads to hyperactivity of cell growth pathways, prominently including mTOR (mammalian target of rapamycin), that contribute to a build-up in cells of toxic aggregates such as progerin (a mutant nuclear cytoskeletal protein), lipofuscin and other cellular debris, triggering formation of senescent cellular phenotypes, which interact destructively with surrounding tissue. Indeed, senescent cell ablation dramatically inhibits physical deterioration in progeroid (age-accelerated) mice. This review explores ways in which oxidative stress creates ageing-associated cellular damage and triggers induction of the cell death/survival programs’ apoptosis, necrosis, autophagy and ‘necroapoptophagy’. The concept of ‘necroapoptophagy’ is presented here as a strategy for varying tissue oxidative stress intensity in ways that induce differential activation of death *versus* survival programs, resulting in enhanced and sustained representation of healthy functional cells. These strategies are discussed in the context of specialized mesenchymal stromal cells with the potential to synergize with telocytes in stabilizing engrafted progenitor cells, thereby extending periods of healthy life. Information and concepts are summarized in a hypothetical approach to suppressing whole-organism senescence, with methods drawn from emerging understandings of ageing, gained from Cnidarians (jellyfish, corals and anemones) that undergo a unique form of cellular regeneration, potentially conferring open-ended lifespans.

- Cellular and molecular features of senescence
  – Introduction: cellular basis for ageing and preliminary intervention strategies
  – Major epigenomic profiles represented in tissue: functional cell categories
  – Progressive shift in epigenomic profiles: a major unifying feature of multicellular ageing
  – Tissue regeneration and cell death: tools for modulation of age-related physical decline
  – Cell stress: an epigenomic rheostat
- Reactive oxygen species (ROS) as mediators of cell death – and cellular phenotype representation in tissue
  – Oxidative stress, cell and tissue damage
  – Reactive oxygen and apoptosis
- Countermeasures to age-associated deterioration
  – Is the senescent cellular phenotype an ‘antagonistically pleiotropic’ ‘longevistat’?
  – Contribution of toxic aggregates to proteinopathies, progeroid syndrome and ageing
  – Hypothetical model of age-associated physical decline and proposed countermeasures
- Conclusions

## Cellular and molecular features of senescence

### Introduction: cellular basis for ageing and preliminary intervention strategies

#### Historical perspective

The phenomenon of physical deterioration with advancing age is a common feature of all multicellular life, with each plant or animal species exhibiting characteristic rates of decline as a normally occurring element of its developmental program. Here, the term ‘apparent’ is used because the existence of a specific genetic mechanism selected by evolution to limit the maximal lifespan of an individual – also termed a ‘longevistat’ – is an ongoing subject of debate [1].

Ageing is a complex and fascinating process that has been the focus of intense efforts to understand and counteract throughout most of human history. Until the 20th century, such initiatives were entirely in the domain of cultural mythology. The most famous of these in Western tradition was the pursuit of a ‘Fountain of Youth’ by the Spanish conquistador Juan Ponce de Leony Figueroa [2].

Advances in medical technology and rapid increases in understanding of cellular and molecular biology have stimulated increasingly sophisticated efforts to define ageing at a level that may allow interventions resulting in lifespan extension. Unfortunately, although enormous gains have been made in the development of therapies for age-associated diseases, such as chronic inflammatory disorders, efforts to decrease rates of physical ageing as a whole have been hampered by an inability to fully characterize the underlying mechanisms of the ageing process. Thus, clear characterization of physical ageing has been difficult due to the lack of a clear case definition that may lead to intervention strategies.

#### Early characterizations of cellular ageing – the ‘Hayflick Limit’

The underlying complexity of age-related physical decline has led many in the scientific community to conclude that the process may represent a general deterioration of cellular function, with insufficient unifying features to allow for definitive interventions. Interestingly, although recent characterization of cellular senescence seems to support such conclusions, ambiguity exists in the interpretation of ongoing studies in this field [3]. This work will be discussed in section I-2 of the present review.

Arguments that ageing is too intricate a process to effectively counteract are diminished to some degree by the implications of studies in the 1960s by Hayflick *et al*. demonstrating the replicative limits to eukaryotic cells in culture [4]. This phenomenon, known as the Hayflick limit, is defined as the average maximum number of doublings a population of normal cells is capable of undergoing before its replicative potential is lost. This maximum *in vitro* doubling capacity was observed to vary, depending on the median lifespan of the species from which cells used to establish the culture were taken. For example, cultures of human foetal cells are observed to double 40–60 times before losing proliferative potential [4], whereas cultures established from mice, a short-lived species, double a maximum of approximately 15 times [5], and cells from Galapagos tortoises, which live well over a century, demonstrate an upward *in vitro* doubling limit of around 110 times in culture [6].

Significantly, it has also been observed that cell cultures derived from patients afflicted with progeroid diseases, in which features of rapidly accelerated ageing are a primary symptom, exhibit far lower Hayflick limits than cells from normal individuals [7]. The strong positive correlation between cell lineage doubling potential (as defined by the Hayflick limit and by longevity of a particular individual) may imply the presence of a normally occurring physiological process acting to limit maximum lifespan within a particular species. This is the definition of a ‘longevistat’ as discussed in a review by Dale Bredesen at the Buck Institute for Age Research, at the University of California in San Francisco [1], which examines evidence for and against the existence of such a process, particularly the contribution of cellular senescence to physical ageing [1]. One implication of the correlation between the Hayflick limit of cells in culture and the median lifespan of the species from which the cells were taken is that, if genetically determined mechanisms for lifespan limitations exist, it may be possible to characterize their underlying features and then to intervene at some point in their normal function in ways that result in lifespan extension.

#### Life expectancy *versus* median lifespan

It is important to distinguish between life expectancy and lifespan. Average life expectancy is the length of time an individual within a population of organisms may be expected to survive when disease, accidents, predation and other environmental stressors are factored in. Conversely, median lifespan is a measure of the time an organism is expected to survive in the complete absence of environmental stressors [8, 9]. The objective of age-intervention initiatives is to make use of cutting-edge technology to extend median lifespan significantly in excess of the normal range for a particular species.

#### Telomere length: correlation between telomere erosion and Hayflick limit *in vitro*

Studies of changes in nuclear chromatin organization occurring during propagation of cell lines have revealed what may constitute elements of a longevistatic process in multicellular organisms. The replicative potential of a particular cell lineage has been observed to correlate with the length of chromosome tips, called telomeres. These structures, which contain non-coding DNA, are shortened each time a cell divides, and after a finite number of divisions, become critically shortened, signalling a cell to stop dividing [10]. The Hayflick limit may thus be defined on a cellular morphological basis as the number of divisions necessary to critically deplete telomeres.

#### Cellular immortality, telomerase activity and telomere length

**Fig. 1:**
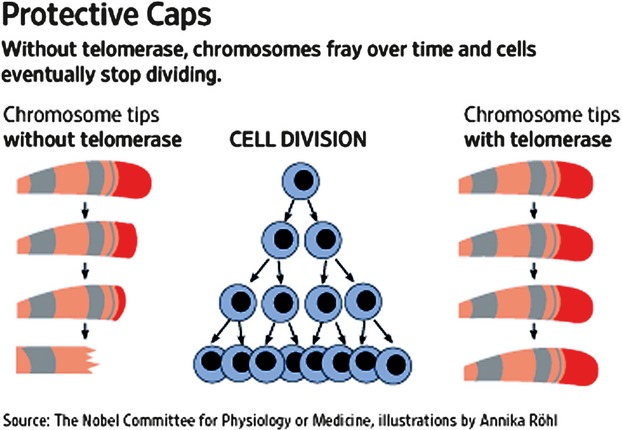
Telomerase and cell division-dependent decrease in telomere length Chromosome telomere length and structural integrity in eukaryotic cells derived from fetal tissue are initially maintained by high levels of telomerase. Activity of this enzyme progressively decreases with each cycle of cell division, resulting in greatly shortened telomeres, decreased organization of chromatin and deleterious effects on cells, including cell division cycle arrest and cellular senescence (Campisi, [3]; Feng *et al*., [13]; Jaskelioff *et al*., [14]).

The aforementioned Hayflick phenomenon may offer insight into strategies for intervention in age-related physical deterioration of an organism based on a major feature of cell division: telomere length. For example, treatment of human fibroblasts *in vitro* with carnosine, a dipeptide antioxidant occurring naturally in vertebrate brain and muscle, decreases telomere erosion rates during cell division and increases the Hayflick limit of treated cultures [11, 12]. An even more potent approach to preserving telomere integrity and maintaining genomic stability of a cell is offered by specifically amplifying expression of telomerase, an enzyme that normally repairs telomeres [13]. The role of telomerase during progressive cell division activity is shown *via* a diagram in Figure 1.

Initially, chromosome telomere length and structural integrity in eukaryotic cells derived from foetal tissue are maintained by high levels of telomerase. The activity of this enzyme progressively decreases with each cycle of cell division, resulting in greatly shortened telomeres, decreased organization of chromatin and deleterious effects, including cell cycle arrest and cellular senescence [3, 13, 14].

Sustained up-regulation of telomerase potentially enables unlimited replicative capacity for a cell [13]. Indeed, telomerase activity is significantly diminished in cells nearing their Hayflick limit both *in vivo* and *in vitro* [3, 13]. Interestingly, multi-organ deterioration in a telomerase-deficient mouse strain that underwent greatly accelerated physical ageing was completely reversed by induction of high activity levels of the enzyme. In these experiments, mice exhibiting physical age near the end of the typical lifespan for this strain were restored to full youthful vigour [14].

Unfortunately, cellular immortalization, including sustained high telomerase activity, is an intrinsic element of carcinogenic transformation of a cell [15–17]. Moreover, as a major feature of virtually all tumour cells is the absence of replication-associated telomere shortening and abolition of the Hayflick limit, a major anticancer strategy currently being pursued is the identification of telomerase inhibitors that restore progressive decrease in telomere length with cell division, thus causing tumour cells to die upon reaching their Hayflick limit.

#### Limits to use of *in vitro* outcome in design of age-intervention strategies

Attempts to identify functional correlations between the Hayflick limit of cultured cells and whole-organism lifespan reveal major paradoxes in efforts to develop strategies for intervention in the ageing process based on outcome of *in vitro* studies of cellular ageing. Extension or abolition of the Hayflick limit by sustained high telomerase activity in a particular cell line has value in ongoing characterizations of ageing on the cellular level, particularly because it may be induced in any modern biomedical laboratory. Nevertheless, clinical use of this phenomenon in age-intervention strategies must be approached with extreme caution as sustained high telomerase activity is a key element of carcinogenic transformation. For this reason, strategies that may extend the replicative potential of cells in a living organism carry a high risk of cancer formation unless countermeasures are employed in such procedures to decrease the risk of carcinogenesis. Approaches for doing this are explored in section III-3 of the present review.

**Fig. 2:**
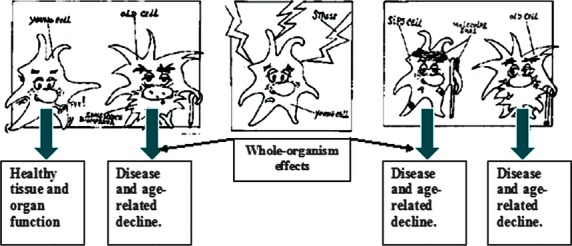
Influence of young undamaged *versus* old or damaged cells on health and disease Young, undamaged cells express products and perform functions that contribute to healthy homeostasis. Old cells and damaged cells that have developed a senescent phenotype (stress-induced prematurely senescent, SIPS) affect surrounding tissue in ways that contribute to age-associated deterioration, including dysregulated inflammation and cancer (Campisi, [3]; Hara *et al*., 2011). **Source: Aging and Stress Group, University of Namur, Belgium.**

Another limitation to fibroblast use of *in vitro* data in the design of animal or human age-intervention strategies is that the behaviour of cells in culture differs substantially from their function as elements of living tissue. *In vitro* models also suffer from weak relevance to whole-organism ageing. For instance, although some investigators have reported that cells taken from elderly human beings exhibit lower replicative potential than those taken from younger volunteers [18], subsequent evaluation of methodologies used to draw these conclusions cast doubt on their validity [19]. An additional pitfall for broad application of *in vitro* data is that cells exhibiting senescent phenotypes have been detected *in vivo*, but without the characteristic shortening of telomeres characteristic of cultured cells at their Hayflick limit [20]. Such cellular phenotypes may arise from healthy cells when subjected to stress that damages their DNA in ways that fail to activate cell death programs and potentially result in carcinogenic transformation. Cells sustaining such damage undergo replicative arrest, preventing their progression into cancers, but also develop a senescent phenotype through the process of stress-induced premature senescence (SIPS), that express products, especially mediators of inflammation, which may impair healthy tissue function, promote carcinogenesis in neighbouring cells and generally contribute to disease- and age-associated whole-organism deterioration [3, 21, 22]. Young, undamaged cells express products and perform functions that contribute to healthy homoeostasis. Old cells and damaged cells that have developed a senescent phenotype affect surrounding tissue in ways that contribute to age-associated deterioration, including dysregulated inflammation and cancer [3, 21]. The influences on an organism of young, undamaged cells *versus* stressed cells that become senescent and aged senescent cells (cells which may have reached their Hayflick limit) are outlined by the illustration shown in Figure 2, below.

If the limitations implied by the aforementioned observations are understood and taken into account in the development of research strategies, cell culture models may nevertheless be used as a very potent tool in designing experiments with expected outcome relevant to whole-organism ageing. Indeed, cell culture models that incorporate SIPS cells and other senescent cellular phenotypes have provided evolving unprecedented understanding of how cellular senescence contributes to age-associated deterioration of tissues and whole organisms [3].

#### Effect of eliminating senescent cells on age-associated pathologies in progeroid mice

In November 2011, investigators at the Mayo Clinic College of Medicine in Rochester, Minnesota reported that selective ablation of senescent cellular phenotypes from progeroid (age-accelerated) mice significantly delayed onset of age-related physical deterioration and decreased severity of established degenerative symptoms in aged animals [23]. These studies utilized a sub-strain of the BubR1 progeroid mouse bearing INK-ATTAC, a drug-inducible transgene, designed to respond to drug treatment by elimination of cells expressing the tumour suppressor protein p16^Ink4^, which is a biomarker for senescent cells and a critical contributor to their emergence through elimination of proliferative capability [23]. When compared with untreated mice, drug-treated animals in which significant attrition of senescent cells had occurred were substantially healthier. Age-related phenotypic parameters that were used as a basis for this observation included: incidence of lordokyphosis and cataracts; mean skeletal muscle fibre diameters measured in 10-month-old mice; physical stamina; body and fat depot weights of 10-month-old mice; fat cell diameters in 10-month-old mice; dermis and subdermal adipose layer thickness of 10-month-old mice; and several other major health-related features that deteriorate with age and at a very high rate in the BubR1 progeroid strain, used as a model in the aforementioned studies [23].

#### Inhibition of accelerated ageing in mice by telomerase reactivation – 2011 Dana Farber studies

**Fig. 3:**
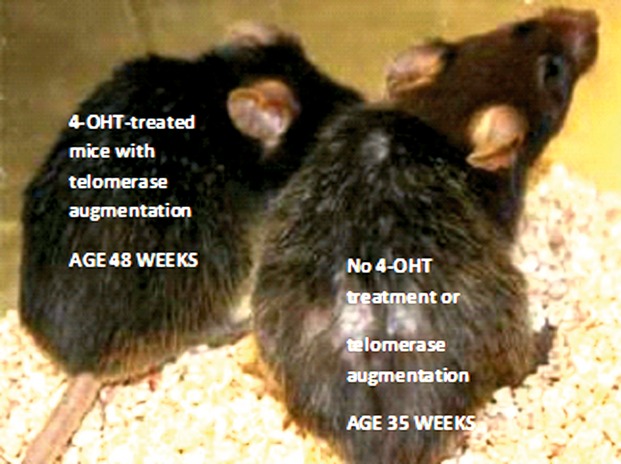
Inhibition of multi-organ deterioration in progeric mice by reactivation of endogenous telomerase expression Telomerase-deficient mice engineered to contain a 4-hydroxytamoxifen (4-OHT)-inducible telomerase reverse transcriptase-estrogen receptor (TERT-ER) allele respond to stimulation with 4-OHT by reactivation of telomerase activity and maintenance of telomere length along with healthy function of tissues and organs. Mice not treated with 4-OHT exhibit low telomerase activity and rapid deterioration of multiple cellular and organ functions expected as an outcome of defective telomeres. Conversely, mice implanted with 4-OHT-releasing pellets experienced somatic telomerase reactivation and significant delay of progeric phenotype onset. Dramatic results of these evaluations are shown below. Here, the 4-OHT-treated, telomerase-enhanced 48 week old mouse on the left, exhibits a physical age apparently younger than the 35 week old non-4-OHT-treated progeric animal. From: Aging Ills Reversed in Mice”. Article by Gautam Naik, The Wall Street Journal, November 28, 2010: Press release, Ronald DePinho laboratory, Dana-Farber Cancer Institute in Boston, MA, USA.”. Photograph by Dr. Mariela Jaskelioff, lead investigator.

Another striking example of how insight gained from *in vitro* studies has contributed to the development of approaches to suppression of age-associated pathologies in animals is provided by the study with age-accelerated mice conducted by Jaskelioff *et al*., described above [14]. This very intriguing research, recently conducted at the Dana Farber Cancer Institute, tested a hypothesis that physical deterioration resembling accelerated ageing in a mouse strain with short, dysfunctional telomeres could be inhibited by systemic amplification of telomerase expression. In these experiments, a transgenic strain of mice was created from telomerase-deficient animals to contain an expression cassette for the enzyme, inducible by the drug 4-hydroxytamoxifen (4-OHT), an oestrogen receptor agonist/antagonist used in therapy for cancer and endocrine disorders. In comparison with sham-treated mice, transgenic animals administered 4-OHT as time-release pellets to reactivate telomerase expression manifested dramatic reversal of the rapid physical deterioration associated with the parent phenotype. Amplified telomerase expression in the transgenic mice restored physical integrity and function of all tissues examined to levels expected in young, healthy animals. These dramatic improvements in prognosis occurred despite the use of mice that exhibited physical deterioration equivalent to extremely aged animals near the end of lifespans expected for the telomerase-deficient strain. Of particular interest was an observation that neural degeneration was completely reversed in animals engineered to contain a 4-OHT-inducible somatic telomerase reactivation element, along with the restoration of smell, reproductive capacity and liver function [14]. The dramatic effect of telomerase reactivation on apparent physical age of mice used for these experiments is shown below in Figure 3.

Telomerase-deficient mice engineered to contain a 4-OHT-inducible telomerase reverse transcriptase-oestrogen receptor (TERT-OR) allele respond to stimulation with 4-OHT by reactivation of telomerase activity and maintenance of telomere length along with healthy function of tissues and organs. Mice not treated with 4-OHT exhibit low telomerase activity and rapid deterioration of multiple cellular and organ functions expected as an outcome of defective telomeres. Conversely, mice implanted with 4-OHT-releasing pellets experienced somatic telomerase reactivation and significant delay of progeric phenotype onset. Dramatic results of these evaluations are shown below. Here, the 4-OHT-treated, telomerase-enhanced, 48-week-old mouse on the left exhibits a physical age apparently younger than the 35-week-old non-4-OHT-treated progeric animal [14].

The relevance of these findings to vertebrate ageing remains to be determined. Despite the enhanced cancer risk likely to be engendered by telomerase-based ‘rejuvenation therapies’, it is tempting to predict that results of the work at Dana Farber reveal a solid mechanistic strategy for intervention in the normal process of ageing. However, at the time of this writing, such a conclusion would be premature, especially since Jaskelioff *et al*. did not report a favourable impact on lifespan as an outcome of their study. Moreover, the extremely high carcinogenic potential of telomerase augmentation makes it unlikely that such an approach to age intervention will become practical for human beings unless and until more effective means of suppressing and/or treating cancer have been developed.

Finally, observations reported by the Dana Farber group were based on a highly specialized progeric mouse strain bred for short telomere length and short lifespan. Hence, their findings should not be extrapolated to other vertebrate models, including human beings, until ongoing studies of telomerase amplification on normal ageing begin to produce definitive data. The status of telomere integrity and telomerase expression in a particular cell species influences its pattern of gene expression (epigenomic profile), which is manifested as cellular phenotype. Here, the term ‘epigenome’ refers to gene expression profiles for any particular cell type, which define its phenotype and homoeostatic role. Epigenomic profiles of cells within a tissue develop in response to interaction between the internal makeup of a cell and its external environment. This, and many other influences, create mosaics of cell phenotype within tissue that ultimately act as major determinants of lifespan, as discussed in section on shifting epigenomic profiles, below.

### Major epigenomic profiles represented in tissue: functional cell categories

#### Lifespan definition and major contributing factors

‘Lifespan’ for any particular animal, plant or cell, may be broadly defined as the average length of time that the organism may resist succumbing to environmental and internal stressors – in the absence of disease, accident or other catastrophic events. The ability of an individual to remain alive and functional is dependent at a fundamental level on the number and distribution of five major functional cell categories, each defined by a unique epigenomic profile. These are described briefly as follows:

- *Regenerative mitotic (stem) cells*: These are cells capable of maintaining tissue integrity by proliferative response to damage, including ‘progenitor’ stem cells, which are specialized types of stem cell differentiated to a degree that allows them to replace a specific type of tissue such as muscle cells or neurons [24].
- *Quiescent post-mitotic cells*: These cells are healthy and terminally differentiated to functional roles in which cell division is not necessary. Examples include nerves [25], heart muscle and other specialized tissue.
- *Transformed cells (cancers)*: Cells that have undergone cancerous transformation represent the greatest drawback to regenerative capacity of a particular type of tissue. A capacity for tissue regeneration has given organisms that have it, such as vertebrates, an enormous adaptive advantage over multicellular life forms in which non-dividing cells constitute the predominant tissue [3]. However, proliferation-capable cells that sustain non-lethal genomic damage in the form of somatic mutations, often propagate this damage by dysregulated clonal expansion [3]. In many cases, such damaged cells exhibit destructive phenotypic characteristics that damage or kill the host. This is the definition of cancer [26].
- *Senescent post-mitotic cells*: Senescent cells are those which are damaged in ways that disable their proliferative ability, but do not cause carcinogenic transformation or cell death. The first stage of cellular senescence is damage-induced cell cycle arrest, also called replicative senescence (RS). Typically, this growth arrest is triggered by stress-induced DNA damage responses (DDR), and also may be induced as a result of effects of telomere erosion – a phenomenon that produces stress-related metabolites [27]. These influences activate tumour suppressor genes, in particular the p53 and pRB pathways, in which damaged DNA and other by-products of cellular stress interact with constitutively expressed cytoplasmic precursors of transcription factors, to form complexes that negatively regulate cell division cycle (CDC) elements and halt proliferation [3]. These cells may remain functional to some degree, depending on the level of damage responsible for abolishing proliferative potential. However, damaged cells in sustained replicative arrest typically undergo a process called ‘geroconversion’, in which hallmark phenotypic characteristics emerge that makes their presence detrimental for the host [3, 27]. Geroconversion is a damage-induced alteration in cellular homoeostasis that causes the emergence of cell forms that adversely impact tissue function. DDR activation resulting from oxidative damage to a cell hyperactivates growth-promoting cellular process, especially the nutrient-sensing complexes mTORC1 and mTORC2, which both include the serine/threonine protein kinase mTOR. These complexes respond to oxidative stress, systemic nutrient level elevation and other cues to maintain high levels of cell growth-related gene expression, especially in the presence of nutrients [27]. As this input of gene products is not counterbalanced by cell division, senescent cells assume a characteristic large, flat or distorted morphology, and often accumulate high levels of pH 6-active beta galactosidase, a lysosomal hydrolase up-regulated by an overload of a cell's degradative machinery as a result of high levels of stress-associated detritus [3, 27, 28]. Senescent cells may also express proteases and inflammatory mediators, such as interleukin-6 (IL-6) that trigger unregulated and pathological inflammatory processes. Accumulation of senescent cells adversely affects their tissue microenvironment, contributing to metabolic syndrome/type 2 diabetes, cancer and many other age-associated disorders [3]. Conversely, deletion of senescent cells delays the onset and reduced severity of age-related pathologies in mice [23]. Further demonstration of rapamycin-mediated mTOR inhibition to enhance autophagic clearance of toxic aggregates and mitigate severity of age-associated disease was reported in October 2011 by researchers at the Institute of Clinical Medicine at the University of Eastern Finland. These investigators demonstrated that the restoration of autophagy by rapamycin-mediated suppression of mTOR activity enhances the viability of human retinal epithelial cells through clearance of lipofuscin deposits [29, 30]. These results further underscore the clinical potential of senescent cells as therapeutic targets. Indeed, as suggested by the authors of the aforementioned report, the results presented suggest the clinical value of a rapamycin-based ophthalmic solution (eye drop) for the treatment for age-associated macular degeneration [29].
- *Cells executing a death program*: Damage to a cell activates several processes that may result in its death. Four major types of cell death have been characterized at the time of this writing [31]:
  - Type I cell death/*apoptosis*: This is a tightly regulated process activated by specific biochemical signalling cues, in which a damaged cell is neatly dismantled in ways that avoid random release of cell contents, thus avoiding tissue toxicity [31, 32].
  - Type II cell death/*autophagy*: Autophagy (or ‘self-eating’) is a process in which the internal components of the cell, in particular, organelles and proteins, are enzymatically degraded. Activation of the autophagic program may not result in cell death, particularly if damage to the cell has not been severe. In fact, under many conditions, it is an adaptive response to stress, particularly starvation, in which degraded cell components may be re-utilized to promote cell survival and restoration of healthy function [27].
  - Type III cell death/*necrosis*: This pathway typically occurs in response to severe stressors imposed on tissue, such as mechanical, chemical or thermal trauma, dysregulated inflammation, microbial challenge and cancer. A major feature of necrosis is the uncontrolled release of cell contents, which may subsequently initiate pro-inflammatory pathways in ways that may become pathological [31].
  - Type IV cell death/‘*necroptosis*’: This process exhibits cell morphological features very similar to necrosis, but also is characterized by biochemical processes that are shared with apoptosis, along with a unique temporal progression distinct from both apoptosis and necrosis [31, 33].

### Progressive shift in epigenomic profiles: a major unifying feature of multicellular ageing

#### Integrating cellular and whole-organism features

Despite the complexity and seeming randomness of the ageing process, studies of its cellular and molecular features have begun to reveal a comprehensive picture of age-associated physical decline that takes into account findings drawn both from work with cells in culture and whole organisms. The health and viability of any multicellular organism at all developmental stages, from embryonic to senescent, is a function of the epigenomic profiles of its constituent cells [34, 35].

The overall physical status of an organism, broadly defined as health and viability, is completely defined by the relative representation of cells in the five major functional cell status categories described above in Section I-2. Each of these categories represents a characteristic pattern of gene expression constituting the epigenome of a particular cell, establishing its phenotype and activity within a particular tissue. Collectively, interacting cellular epigenomic profiles are manifested as tissue and whole-organism health status. Ageing may therefore be defined as the progressive shift in epigenomic profiles of cells that collectively make up an individual, and age-related physical deterioration occurs as a function of how far these shifts move an individual away from steady-state homoeostatic equilibrium (representing ideal health).

Individuals with an elevated fraction of cells in a healthy quiescent post-mitotic state (Category II) are likely to exhibit balanced homoeostasis and good overall health. Likewise, organisms undergoing significant growth, in which large numbers of dividing cells (Category I) are adding healthy tissue, may exhibit robust viability, particularly if the cell growth is part of an existing developmental program representing the transitional stages of early life. The viability of an organism with high representation of Category I cells may nevertheless be low if high rates of cell proliferation are occurring in response to trauma. In such cases, elevated mitotic activity may be an adaptive response, with the potential to restore a physiologically compromised individual to its optimally healthy state.

The presence of category III cells (cancer) is an obvious and typically lethal disadvantage, even when the percentage representation of such cell species is low. Signalling pathways that result in geroconversion into senescent cellular forms (Category IV) effectively block carcinogenic transformation. Nevertheless, age- or trauma-related tissue build-up of senescent cellular phenotypes creates progressively toxic tissue microenvironments that favour transition to cancer and other degenerative processes [3]. The overall deleterious influence of senescent cells on whole-organism viability is significant in normal ageing. Cells that have undergone replicative arrest as result of both stress-induced damage and telomere erosion (which constitutes a special kind of stress, as will be discussed below), develop into senescent forms that accumulate over time and become major contributors to age-associated pathologies such as atherosclerosis, stroke and other chronic syndromes characterized by dysregulated inflammation [3, 13, 27].

#### Role of cell death programs in tissue cell phenotype representation

Of major interest in the context of the present review is the relative representation of Category V – that is, cells that have been activated to execute signalling pathways leading to death *via* apoptosis, autophagy, necrosis or necroptosis. Each of these four major modes of cell death is potentially useful in counteracting normal age-associated physical decline and maintaining the representation of cellular phenotypes that maximize an individual's retention of youthful characteristics. The rate and magnitude of cell death occurring in a particular tissue establishes the distribution of cellular phenotypes and effectively defines virtually all aspects of an individual's physical makeup at any given developmental stage and environment. It is therefore likely that strategies for intervention in normal ageing will become increasingly focused on methods for skewing representation of cells towards increased percentages of healthy, quiescent forms, and where needed, healthy replicative or regenerative types: cell phenotype categories I and II described in section I-2 above) respectively. At the time of this writing, therapeutic methods that skew cells in a particular tissue towards desired phenotypic representation patterns have not been used specifically to target ageing in human beings. Phenotypic skewing by selective induction of death, *versus* survival programs within a tissue may nevertheless become an increasingly important tool in life extension strategies with increased understanding of molecular mechanisms leading to cell death. An exploration of how oxidative stress contributes to activation of the apoptotic death program is provided in Part II of the present review.

#### Developmental shifting of cellular phenotypic representation in tissue

**Fig. 4:**
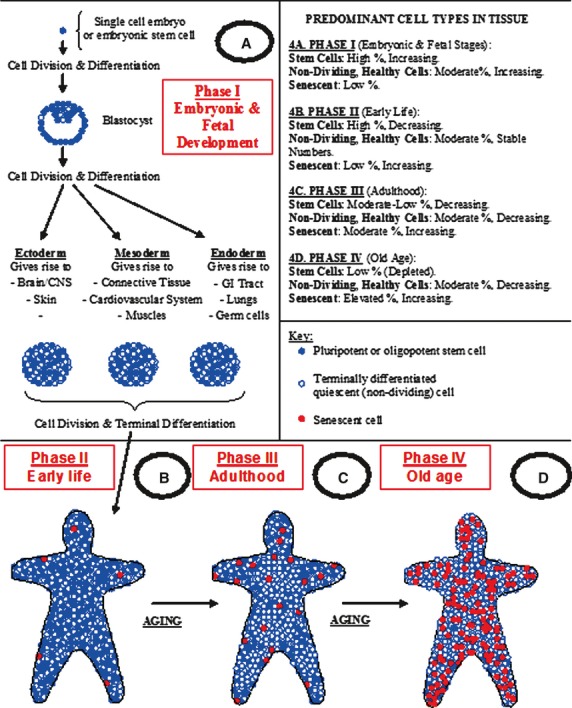
Development/age-related shift in tissue representation of functional cell types.

Eukaryotic multicellular organisms begin as an undifferentiated pluripotent stem cell, normally in the form of a single cell embryo, which may proliferate with little differentiation to form a peri-implantation embryo, or blastocyst. The component cells then undergo further proliferation and differentiation to form an organism's three classes of progenitor tissue: endoderm, mesoderm and ectoderm. During early life, each tissue contains proliferation-competent progenitor cells (functional category Type I) in percentages that are elevated relative to later developmental stages. These ‘oligopotent’ stem cells are capable of terminal differentiation into specific types of tissue and serve as a pool of reserve tissue to replace quiescent post-mitotic cells (functional category II) as they become depleted through stress-induced attrition. Cell damage which fails to move a cell into functional category V through activation of a death program may cause genetic changes, causing transition to functional category III (cancer). Vertebrate eukaryotes have evolved a major anticancer adaptive program followed by damaged cells, which causes their conversion into senescent phenotypes (category IV), which are incapable of proliferation, but remain alive. Progressively decreasing percentages of quiescent and regenerative cells in tissues during adulthood through old age are paralleled by increased representation of senescent cells [3]. At the time of this writing, although no direct mechanistic correlations between cellular senescence and whole-organism ageing have been established, the known effects of senescent cells on resident tissues provide the basis for a compelling hypothesis. Specifically, a possible explanation for physical decline that accompanies the chronological age of an organism is that progressively elevated numbers of senescent cells in each tissue adversely affect tissue integrity and function in ways that are manifested as senescent whole-organism phenotypes. Approaches to testing of this hypothesis in ways that may lead to strategies for counteracting normal ageing are discussed in section III-3 of the present review. A conceptual picture of an age-related shift in representation of functional cell categories is shown below, in Figure 4.

### Tissue regeneration and cell death: tools for modulation of age-related physical decline

#### Stem cell-mediated tissue regeneration strategies

Approaches that may be used in skewing modes of cell death in ways that favour youthful representation of functional cell categories in tissue will be discussed in section III-3 of this review. Below in the present section, some of the approaches for augmenting a representation of Category I, healthy replication-competent (stem) cells will be considered. One treatment that relies on such a process, bone marrow transplantation (BMT), has a well-established track record in clinical medicine. This method, which regenerates haematopoietic tissue by supplementation with allogeneic stem cells, is discussed below.

#### BMT – an early, prototypic stem-cell therapy

The most widely used form of cellular regenerative therapy that relies on repopulation of a tissue niche with Category I (mitotic) cells is BMT. BMT, for reasons discussed below, has features that allow it to serve as a very early prototypical model for stem cell-based rejuvenation therapy. Nevertheless, BMT has never been used specifically as an anti-ageing measure, and in its present form, it is not likely to have direct application in any age-intervention strategies. BMT is a drastic, invasive and painful treatment, typically used for patients afflicted with neoplastic lymphoproliferative disorders (including leukaemias) who have proven refractory to chemotherapy, radiation and other commonly used anticancer regimens. Nevertheless, observations of the behaviour of transplanted haematopoietic stem cells (HSC) help establish guidelines for transplantation of a wide range of other progenitor cell types. For example, recent studies demonstrating the ability of HSC from young mice to suppress cell senescence-related biomarkers and rejuvenate renal tissue in aged mice [36], suggest that BMT may provide a therapeutic paradigm of potential use in the design of clinical protocols for counteracting physical ageing.

Preparation of a patient for BMT may involve complete ablation of bone marrow, using whole-body radiation or chemotherapeutic agents such as cyclophosphamide and busulfan. Although on a case-specific basis, a ‘non-myeloablative’ protocol that leaves recipient bone marrow in place may be used. The reduced level of trauma as a result of treatment with this latter procedure is offset somewhat by an increased risk of cancer relapse. A patient is then administered either autologous or allogeneic cell preparations, which have high percentages of HSC. These may be obtained from donor bone marrow or from the peripheral blood of either a donor or the patient by a process called apheresis, which extracts HSC from blood. The therapeutic effect depends upon the ability of transplanted HSC to replace a patient's original haematopoietic tissue and provide the individual with a self-renewing source of progenitors for each of the haematopoietic lineages.

Refinements in the clinical use of BMT have resulted in steady improvements in its efficacy during the late 20th and early 21st centuries. Nevertheless, the adverse effects associated with this procedure underscore the dangers associated with the use of stem-cell transplantation in broad clinical venues. The major complications of BMT include several life-threatening conditions that develop as a result of the method of applying this treatment, and also as a consequence of stem-cell behaviour. The general immunosuppression that accompanies the destruction of recipient bone marrow, along with the effects of immunosuppressive drugs, leaves a patient vulnerable to opportunistic infections and sepsis. BMT recipients are also highly susceptible to new malignancies that may arise from carcinogenic influences on host tissue of marrow-ablative treatments. BMT carries an additional risk of tumour evolution from transplanted HSC, as placement in their new environment may stimulate dysregulated proliferative responses, including carcinogenesis.

Another major obstacle to successful BMT currently faced by healthcare providers is the requirement for a close human leucocyte antigen (HLA) tissue match between donor and recipient to avoid graft-*versus*-host disease (GVHD), an often fatal syndrome resulting from immune attack on host tissue by transplanted donor leucocytes [37]. Typically, a prospective BMT recipient's siblings are screened for suitability of donors and failing this, the search is widened to unrelated persons. Nevertheless, at the time of this writing, a large percentage of patients requiring allogeneic HSC are unable to identify matching donors [38].

#### Mesenchymal stromal cell influence on differentiation and immunogenicity in bone marrow cultures

A rapidly evolving and very exciting strategy for increasing safety and efficacy of BMT, with profound implications for sustained regenerative capacity of all tissues, is the use of mesenchymal stromal cells (MSC), which were first identified as a subpopulation within the bone marrow that may act as a ‘feeder’ cell layer to sustain HSC function *in vitro* [39]. A major unique property of MSC is ‘multi-potency’. That is, their interaction with tissue culture surfaces stimulates differentiation into a wide variety of functional cellular phenotypes, exhibiting ectodermal, endodermal and mesodermal epigenomic expression profiles [39, 40]. The potential for therapeutic use of such phenotypic ‘plasticity’ by MSCs is augmented by their negligible immunogenicity [41]. This property enables MSCs to evade alloreactive cell recognition, thereby allowing their clinical use without the requirement for donor–recipient HLA tissue match [39]. MSC are also observed to down-regulate T lymphocyte immune activity [42], a property which offers enormous potential for their application in the prevention and management of tissue damage resulting from hyperactive inflammatory processes. Indeed, host MSC are observed to reduce the risk and severity of GVHD in BMT patients through the inhibition of T cell-dependent pro-inflammatory processes [43], the recruitment of donor HSC to host bone marrow, enhancing their engraftment and promoting haematopoiesis [39].

The foregoing observations reveal host-derived MSC to be a critical component for bone marrow regeneration and maintenance. However, the evolution of MSC-based preventive medicine and therapies will require sustainable sources of allogeneic MSCs. Fortunately, MSC lines derived from umbilical cord blood may be functionally expanded *in vitro* to promote engraftment of progenitor stem cells capable of regenerating a wide variety of tissues [39].

Placenta-expanded MSC product-1 (PLX-1) aids in engraftment of HSC derived from umbilical cord blood (UCB). The first mainstream medical application of UCB MSC was made by Pleuristem Therapeutics of Haifa, Israel, which in 2008 obtained U.S. F.D.A. clearance for Phase I clinical trials of a product called PLX-1 cells, which are placental-derived MSCs expanded *ex vivo* and stored frozen before being administered to patients by i.v. injection [39].

#### Relevance of PLX-I and related MSC to age intervention

A major objective of Pleuristem and other parties developing allogeneic cell lines capable of enhancing representation of regenerative cells in a particular tissue is to broaden the range of tissues into which products such as PLX-I may be engrafted. Progressive refinement of this technology may ultimately allow a sustained regimen of treatments to continually regenerate a comprehensive approach that facilitate achievement of this objective. A major outcome may include maintenance of a healthy and balanced tissue homoeostasis for very long time periods – which could be manifested as a prolonged lifespan. Such an outcome would also require simultaneous protection against cancer and deletion of senescent cells. Consideration of factors contributing to these objectives is discussed below.

#### Activity of mTOR in bone marrow

BMT offers a particularly good model for initial efforts to restore the regenerative capacity of a representative tissue and prolong the time period that it is stable and functional. Elevated representation of senescent cells in the bone marrow of a recipient can adversely affect the capacity of stem cells introduced *via* BMT to restore function and viability of haematopoietic [27]. As previously described, a major contributor to the development of the senescent cellular phenotype is hyperactivation of nutrient sensor and growth pathways, in particular mTOR and its derivative complexes mTORC1 and mTORC2[3, 27, 28]. These findings suggest that age-intervention strategies are likely to include pharmacological inhibition of mTOR activity, an approach that will be discussed in Part 3 of the present review.

In healthy bone marrow, or marrow successfully restored to functional capacity by BMT, a pool of self-renewing ‘progenitor’ HSC (Category I in section I-2 above) are distributed throughout the tissue and maintained in a metabolically active quiescent state which, in response to physiological cues, is capable of progression into the cell cycle and production of blood components. During healthy HSC proliferation, cell mass increases due to up-regulation of mTORC1 signalling keeps pace with cell division and cells of normal size and function are produced. However, in a tissue environment which favours potentially pathological levels of cell growth signalling, mTORC activity becomes hyperactivated as a result of the critically decreased availability of the major negative regulators of this enzyme complex, resulting in the exhaustion and depletion of HSCs. Dysregulation of mTOR is also observed to modulate the activity of the enzyme in ways that promote enhanced mitochondrial biogenesis, with resulting increases in oxidative stress [44] and decreased differentiation of HSC into cell forms that contribute to normal homoeostatic function. By contrast, the capacity of HSC to regenerate functional blood cell lineages is dramatically increased by inhibition of mTOR. As mTOR is a negative regulator of autophagic processes, inhibition of this enzyme increases autophagy, resulting in clearance of toxic debris and increasing autophagy and suppression of the emergence of senescent cellular phenotypes [44]. These aforementioned outcome taken in context with demonstration of the rejuvenating effects of BMT from young mouse donors on aged mouse kidney [36], underscore the value of BMT as an example of how stem cells might be applied as a countermeasure to age-associated physical decline.

#### Contribution of cell growth-promoting mechanisms to cellular senescence

Hyperactivity of growth-promoting processes such as mTOR activity is a major contributor to the toxicity of tissue environments that contain a high representation of senescent cell types [3, 44]. This, and other features of senescent cells, underscores the requirement to counteract the influence of these cells – and if possible eliminate them as an integral element of age-intervention strategies. Any approach to rejuvenation-oriented skewing of functional cell types in a tissue must consider how dysregulated mTOR hyperactivity and other adverse influences of senescent cellular phenotypes, such as their pro-inflammatory and carcinogenic effects [3], may affect healthy cells.

#### Influence of senescent tissue environment on stem-cell function

The effect of the tissue environment on stem-cell function is explored in an elegant study by Irina Conboy *et al*. in an investigation of progenitor stem cells for muscle, called myogenic satellite cells (2005). This investigation revealed that myosatellite regenerative function was significantly impaired in tissue from young healthy mice that had been grafted into the senescent environment of aged animals. This investigation further revealed that the myosatellite regenerative and functional capacity was restored when tissue taken from elderly donors was placed in younger recipients [45].

#### ‘Parabiotic pairing’

A fascinating corollary experiment by these investigators demonstrated that the rejuvenating effect of younger tissue on stem cells from elderly individuals could be mediated by humoural factors. These procedures made use of a technique called ‘parabiotic pairing’, in which the circulatory systems of two syngenic mice were linked, exposing the aged animal to the environment of the younger one and *vice versa*.

Restoration of youthful competence to myosatellite cells in aged mice was determined to have occurred due to reactivation of a signalling pathway called ‘Notch’, which declines in strength with age, but is restored in the elderly member of a parabiotic pair through mechanisms yet to be characterized. These findings, and analysis of the effect of senescent tissue environment on HSC, clearly suggest that the tissues of elderly individuals cannot be restored to youthful vigour by simply introducing a new supply of progenitor stem cells. It is likely that the functionality of these cells would be rapidly degraded by the influence of resident senescent cellular phenotypes. A possible countermeasure to senescent cell influence might be the incorporation into genomes of progenitor stem cells, to be administered therapeutically, of expression cassettes engineered to produce activators for critical pathways such as notch. The expression of such a signalling molecule might be made drug-inducible, as was accomplished by the team at Dana Farber, who constructed a 4-OHT-inducible cassette to increase telomerase production in mice [14].

At present, however, such a strategy is highly speculative. Moreover, safety issues make such an approach extremely challenging. For example, inflammatory cytokines (including IL-6) which may be up-regulated in senescent cells by an anti-apoptotic protein HMGB1 are known to stimulate Notch signalling in ways that augment breast cancers [46]. These observations underscore the need to consider how a senescent environment may affect stem cells introduced as an element of rejuvenation therapy – and how some future novel class of stem cell programmed to express signalling pathway mediators may endanger resident tissue.

#### Altering tissue cellular phenotypic ratios by varying oxidative stress dosage: induction of apoptotic responses *versus* senescence

Future strategies that are able to impart sustained youthful vigour to whole organisms will almost certainly include methods for selectively eliminating cell types that detract from the functional efficiency of component tissues. In a broad sense, external stress applied to cells, both *in vivo* and *in vitro*, achieves this purpose. Varying the intensity of a wide range of stressors to which a tissue or organism is exposed has been observed to reproducibly shift the percentage representation of senescent cells, with those executing each of the four major pathways to death (apoptosis, necrosis, necroptosis and autophagy). Relatively low stress levels favour predominance of senescent forms, followed by the induction of apoptotic programs at higher levels, transitioning to necrosis when stress levels become extreme [47].

#### Induction of autophagy and necroptosis

Cells which do not respond to oxidative stressors by senescence or apoptosis may die *via* ‘necroptosis’, or undergo autophagy, which can result in either cell death or survival under improved conditions of cellular homoeostasis [31]. Cells are influenced to respond to various stress conditions by a particular death or survival pathway depending on the presence of certain pharmacological agents, or as part of unique pathologies that may favour skewing of cellular activity patterns into one of the aforementioned stress-response modes [31]. Cells may sustain a very wide variety of stressors, all forms of which cause activation of DDR mechanisms, resulting in cell cycle arrest and either senescence, repair and survival, activation of a death program or carcinogenic transformation [3, 27]. Typically, DDR activation occurs as a result of a reaction between DNA and reactive oxygen molecules that cause strand breaks and production of DNA degradation products which trigger cell cycle arrest through induction of the ‘Gatekeeper’ tumour suppressive proteins p53 and pRB1/p16. This either directly inhibits the transcription of genes needed for mitosis, or promotes the expression of other protein inhibitors such as p21, the expression of which is driven by an interaction of p53 with the p21 element's promoter [3]. DDR activation hyperstimulates cell growth mechanisms such as mTORC, which in turn promote the development of senescent phenotypes and toxic tissue microenvironments [27].

### Cell stress: an epigenomic rheostat

#### Cellular consequences of DDR activation

The major common feature in virtually all influences that cause stress-induced epigenomic changes is an interaction between a cell's DNA and reactive oxygen molecules produced by the stressor – with resulting activation of DDR processes and conversion of the cell from one of the phenotypic categories described in Section I-2 above into another [3, 27, 31]. Some stressors activate DDR without DNA damage [48]. Therefore, within certain limits, manipulation of external stress levels (including mild thermal stress, cold and other influences that avoid significant cellular damage) potentially provides a general approach for shifting epigenomic profiles in ways that promote tissue and whole-organism vitality. By the foregoing analysis, if stress levels delivered to a particular tissue can be varied with some degree of precision, then differential application of such stressors constitutes a biological rheostat. Ideally, such an approach might be used to reproducibly vary the percentage representation of certain cell phenotypes, much as an electrical rheostat is used to adjust the level of analogue signals on (for instance) a light dimmer, or audio volume control.

#### Oxidative stress-mediated influence on components of the nuclear cytoskeleton: relationship with progeria and normal ageing

An example of how altering tissue oxidative stress levels may be used to manipulate ageing-related cellular signalling is provided by the outcome of the study that demonstrated how up-regulation of oxidative stress by the drug 6-hydroxydopamine alters phosphorylation patterns of the nuclear cytoskeletal proteins lamin A/C. Altered phosphorylation changes the interaction between this lamin and heat shock protein-90 (hsp-90) in human neuroblastoma cells in ways that correlate with the role of lamin A/C in age-related cellular and tissue changes [49]. These results are particularly significant in the context of the role of progerin, a mutant variant of lamin A, which adversely alters nuclear scaffolding, causing disruption of cellular homoeostasis, cell cycle arrest and rapid geroconversion into senescent cellular phenotypes [27].

High expression of progerin has been identified as the cause of Hutchinson-Gilford progeria syndrome (HGPS) [50]. HGPS-afflicted human beings exhibit rapidly advanced rates of physical ageing, dying at approximately 13 years of age from the same spectrum of disorders, such as cardiovascular syndromes, which are typically the cause of death in elderly human beings [50]. Sporadic expression of a cryptic splice variant for lamin A also results in progressive accumulation of the defective protein in cells of healthy individuals, thus allowing the use of progerin as a reliable biomarker of ageing [51, 52]. Therefore, the aforementioned studies by Nakamura *et al*., demonstrating the capacity of oxidative stress to act through nuclear lamin to affect age-related cellular biomarkers [49], increase the scope of potential contribution of oxidative cellular processes to progerin-related cellular and whole-organism senescence. Cells of the cardiovascular system are, for reasons not completely understood at the time of this writing, particularly prone to disruption of cellular homoeostasis due to build-up of progerin and other toxic debris [50]. This phenomenon may at least partly explain the observation that cardiovascular disease risk factors, including hypertension, hyperlipidaemia, diabetes, insulin resistance, atherosclerosis and heart failure, increase dramatically with age as contributors to mortality among the elderly [53]. The effect of these influences may be mitigated somewhat by short ischaemic periods interspersed with brief periods of reflow just prior to reperfusion of cardiac tissue, which is a process called ‘ischaemic post-conditioning’ [54]. Characterization of mechanisms by which cellular debris disrupts cardiovascular cell function in ways that exacerbate risk factors and lead to disease is an area of ongoing intensive research.

#### mTOR, autophagy and oxidative stress-related effects on geroconversion: progerin-mediated

Research ongoing at the time of this writing has shown that the progerin-mediated geroconversion of both HGPS and normal human cells may be prevented and under some conditions reversed by induction of autophagy through suppression of mTOR with rapamycin, an mTOR-inhibitory drug [55]. In normal cells, mTOR activity suppresses autophagy thus allowing progerin and other cellular debris to create oxidative damage resulting in cell cycle arrest and geroconversion to senescent cell phenotypes [55]. For this reason, inhibition of mTOR activity allows autophagic processes to occur at elevated rates, causing ‘cellular housecleaning’ that clears progerin and other mis-functional metabolic products to an extent that enhances healthy cell function [27]. mTOR activity is strongly stimulated by oxidative stress and inhibited by antioxidants [56, 57]. The ability of rapamycin to inhibit mTOR and thus facilitate autophagic clearance of senescence-inducing protein aggregates is potentially a very powerful tool in age intervention. Nevertheless, the high toxicity of the drug makes it impractical for its administration at dosages needed for sustained suppression of senescent cell accumulation in a particular animal [58]. Despite that, a strategy that makes use of a principle called ‘phytochemical synergy’ may allow for substantial reduction in its effective dosage – and thus toxicity. These studies demonstrated that non-toxic phytochemicals extracted from *Ginkgo biloba* were capable of modulating calcium metabolism in ways that allowed the immunosuppressant FK506 to mediate powerful antiarrhythmic effects in a rodent heart model at significantly lower dosage than FK506 alone [59]. Similar strategies pursued in ongoing studies at University of Debrecen may allow for reduction in the effective dosage and thus toxicity of rapamycin.

#### Cardiovascular ageing: why age-related physical decline most often involves cardiovascular tissue

The effect of progerin and related protein aggregates in various cell types yield insight into the reason cardiovascular pathologies are a widely prevalent common feature of age-related physical deterioration. Mechanistic insight is provided by an observation that human vascular cells express progerin. These cells are particularly susceptible to disruption of cellular homoeostasis and deterioration in healthy function [50]. Thus, in normal ageing, it is likely that increased representation of cells bearing toxic protein aggregates by all tissues will impact the cardiovascular system earliest and most severely.

Recent studies by Rakesh Kukreja and colleagues at Virginia Commonwealth University revealed that rapamycin constitutes a particularly powerful countermeasure to cardiovascular ageing. Using a murine model of myocardial ischaemia-reperfusion (I/R) injury with isolated hearts in a Langendorff perfusion system, along with parallel *in vitro* experiments, these investigators demonstrated the ability of rapamycin to significantly protect whole hearts against I/R injury, and further showed that the drug suppressed simulated ischaemia-reoxygenation (SI/RO) damage to primary cardiac myocytes. These studies revealed that the underlying mechanism included activation of JAK2-STAT3 transcription factors, with a subsequent cascade of cardioprotective signalling events, including phosphorylation of ERK, STAT3, eNOS and glycogen synthase kinase-3ß in concert with increased pro-survival Bcl-2 to Bax ratio [60]. These outcome provide a basis for improved strategies for prevention and treatment of cardiovascular disease in ways that directly counteract degenerative changes occurring during the normal process of ageing.

The foregoing reports describe how oxidative stress may promote accumulation of senescent cells through increased mTOR activity and by adverse effects on cytoskeletal components such as lamins. A picture emerges from the aforementioned, suggesting a strategy for inhibition of age-related physical decline by promoting levels of autophagy sufficient to enable ongoing disposal of toxic cellular products such as progerin, but not high enough to cause significant autophagic cell death. A major mediator in achieving such a balance is mTOR, the variation in oxidative stress in particular, as a primary tool for manipulating mTOR activity.

It is important at this point to qualify the above analysis by emphasizing that such an approach could almost certainly never be finely tuned enough to use it as a primary mechanism of rejuvenating tissue – or a whole organism. The use of dietary antioxidants represents a popular, but poorly understood application of redox status adjustment that is occasionally touted as ‘anti-ageing’ therapy. This notwithstanding, selective use of antioxidants and possibly other pharmacological agents will almost certainly have value in optimizing conditions for the activity of stem cells that might be used for sustained tissue regeneration approaches. As the purpose of this review was to establish a solid molecular biological and cellular basis for developing a general life extension strategy, Part II of the paper is a comprehensive review of cellular redox sources of reactive oxygen and how these molecular species interact with cell components to either promote survival or activate a death pathway, with a focus on apoptosis.

## Reactive oxygen species (ROS) as mediators of cell death – and cellular phenotype representation in tissue

### Oxidative stress, cell and tissue damage

As described above, oxidative stress plays a pivotal role in the age-related deterioration of cells, tissues and whole organisms. Numerous other human pathologies are characterized by oxidative tissue damage. The most damaging of these are dysregulated inflammatory processes in which symptoms resulting from tissue injury are substantially mediated by highly reactive oxygen-containing molecules that may be elevated systemically and/or locally at the site of tissue injury. ‘Oxidative stress’, which broadly refers to the collective effect of these processes, may be caused by a wide range of influences, including disease, drugs such as doxorubicin, mechanical or thermal injury, and exposure to radiation or toxic chemicals [61].

### Reactive oxygen and apoptosis

#### Apoptosis

Apoptosis is a tightly regulated ‘suicide’ program, involving sequential dismantling of a cell's structure proceeds according to a series of steps designed to minimize the release of potentially harmful products and provide for their efficient disposal. Superoxides and related species have been implicated as major mediators of several forms of apoptosis. For example, exposure of a cell to ionizing radiation will cause the formation of high concentrations of hydroxyl (°OH) radicals as a result of radiolytic breakdown of water. These react readily with DNA, protein and lipids to form nucleotide adducts and lipid peroxides. The resulting damage activates the cell's apoptotic program [62]. A major objective in strategies to inhibit age-related deterioration in tissue function is to identify critical redox signalling pathways that may be used to drive damaged cells into apoptotic death as opposed to carcinogenesis or senescence.

#### Carcinogenesis *versus* apoptosis

The role of ROS in both of these processes presents an apparent paradox: in each case, elevated levels of ROS favour departure from normal cellular homoeostasis, either to apoptotic death or tumour formation. At the time of this writing, not enough is known about the dynamics of either process to define the specific interactions leading to one end or the other. One major juncture at which a cell may either turn cancerous or die by apoptosis seems to be dependent on the fate of PARP, which, responding to DNA broken ends acts to promote proliferation and (presumably) survival of a cancerous line.

During apoptosis, PARP undergoes cleavage by the apoptosis-associated protease CPP32/YAMA [63, 64, 65]. Presumably, if enough CPP32-mediated destruction of PARP occurs before the action of PARP tips the balance to proliferation, apoptosis will occur, abrogating a potential tumour. CPP32 is a critical element in this protective process. If in fact superoxide-mediated inhibition of ICE occurs, then PARP inactivation would be considerably reduced or absent in a pro-oxidant tumour. A possible mechanism of oncogenesis involving superoxide-mediated inhibition of CPP32 proteolysis of PARP can be inferred from the known roles of both elements. CPP32/YAMA is a member of a family of macromolecules that include the Interleukin-1ß-converting enzymes (ICE), which are cysteinyl aspartate-specific proteases, most commonly referred to as caspases, with activities that constitute the core processes of apoptosis [66]. Two broad classes of apoptosis-associated ICE have been characterized based on their major role in the apoptotic process. These include the ‘initiator’ ICE forms, which trigger apoptosis by cleavage of effector caspases, thereby activating them to cleave substrates that include cell structural components.

#### Pro-oxidant degradation of antioxidant defence

All cells studied thus far express very low constitutive levels of apoptotic caspases and up-regulate the enzyme only when required for apoptosis [67–70]. By contrast, other compounds bearing reactive -SH groups are present at high concentration – including ROS scavengers such as glutathione (GSH), which are present at millimolar levels [71]. How, then, might a situation arise in which sufficient concentrations of both O_2_^−^ and caspases are present such that the former might inhibit the latter by thiol oxidation, either at the catalytic Cys or other Cys residues?

Such a phenomenon might develop as a result of defective O_2_^−^ removal. As impaired function of superoxide dismutase (a specific scavenger of O_2_^−^) is a hallmark of a wide variety of tumours [72], high levels of superoxide production are expected, resulting in oxidative stress and diminished antioxidant protection as a result of a decreased concentration of GSH.

#### GSH as a pro-oxidant agent

This reduction in available GSH is a hallmark of the pro-oxidant state [73], and may arise as a result of the reaction between high concentrations of O_2_^−^ with GSH. Under normal conditions (as previously stated), O_2_^−^ is dismuted primarily by SOD and does not interact significantly with GSH. However, when defects in SOD processing of O_2_^−^ allow its accumulation, it will react with GSH in a chain-propagating process which causes GSH to amplify oxidative stress rather than dampening it [71]. Moreover, because O_2_^−^ is actually regenerated during such a process, it is free to react with additional GSH or other cellular components.

#### Apoptotic caspases regulate superoxide-induced apoptosis

The observations described above suggest that the phenomenon of superoxide/thiol interaction, particularly where impaired SOD activity exists, substantially deplete GSH [64, 74, 75], and possibly thioredoxin, leaving other cellular elements open to O_2_^−^ mediated damage. Many potential targets exist, including (but by no means exclusively) -SH and other reactive groups on caspases and their precursors. Six major arguments may be offered for role of caspase as a superoxide-sensitive target in abrogation of FAS-mediated apoptosis under pro-oxidant conditions [76]. Each argument is briefly summarized below:

1. Superoxide-sensitive cellular components must be essential for FAS and TNF-dependant but not perforin/granzyme-mediated apoptosis [77, 78].
2. The element which is inactive must be a ‘master switch’, that is, its function is absolutely necessary for apoptosis.
3. Elevated levels of O_2_^−^ confer resistance to FAS-mediated apoptosis, and reducing these levels restores FAS sensitivity [79]. Hence, either O_2_^−^ itself or some product secondary to its presence inactivates one or more elements critical for apoptosis. ICE (family) protease activity is critical for apoptosis and ICE inhibitors also inhibit apoptosis [79].
4. Additional evidence that ICE family proteases constitute a physiological ‘master switch’ for apoptosis, is provided by the observation that CrmA, a general serpin inhibitor of the ICE family will inhibit almost all forms of apoptosis. Upon comparison of the perforin/granzyme, FAS and TNF pathways, an ICE protease (MACH) is vital for the two latter, but not the first pathway. Moreover, Boldin *et al*. demonstrate that specific inhibition of MACH will abrogate apoptosis [80–83].
5. MACH is the most upstream non-membrane-bound element of the FAS (and) TNF pathways.
6. While it is true that concentrations of *active* ICE family proteases are present at extremely low levels, their preformed precursors are present at constitutively higher levels. The reaction of superoxide with solvent-exposed thiols (or other groups) on these precursors might result in inhibition of active ICE formation, or the assembly of poorly functional ICE.

#### Superoxide-sensitive non-thiol groups on ICE and other elements of cellular apoptotic pathways

Thiol groups on ICE proteases or its precursors are suggested as the most likely targets for superoxide-mediated inactivation. However, there is no reason that other interactions between superoxide and components of ICE (or precursors) might result in an inhibitory effect. As an example, physiological levels of hydroxyl radical (°OH) are observed to react with tyrosine on some proteins, resulting in some covalent modification (here the notation ‘°OH’ represents a hydroxyl group with a single unpaired electron [84]).

#### Superoxide-mediated manipulation of cell fates

Caspase-superoxide interactions provide a paradigm for controlling entry of a cell into a particular death program (apoptosis in the above summaries). In addition, as depletion of GSH by superoxide correlates with an onset or increase in superoxide-mediated caspase inhibition, pharmacological alterations in the availability of this molecule might be used as an additional measure of control for initiation of the apoptotic program. Many other features of the ROS-driven cell death programs are potential targets for pharmacological control of cell death and modification in tissue composition.

## Countermeasures to age-associated deterioration

### Is the senescent cellular phenotype an ‘antagonistically pleiotropic’ ‘longevistat’?

#### Senescent phenotype: a cellular paradox

The preceding section provides an overview of how ROS interact with critical regulatory molecules of the apoptotic pathway to induce cell death. Caspases are a representative example. Many other molecules, such as PARP, may also emerge as targets for modulation as part of age-intervention strategies. However, the optimal use of this information in skewing cellular phenotypic representation to compositions that favour indefinitely sustained vitality of tissues – and whole organisms – will require an improved understanding of cellular senescence. The existence of senescent cells presents a fascinating paradox. These cells are an example of an ‘antagonistically pleiotropic’ trait. These confer an advantage early in life, but decrease the capacity for survival with increasing age [22].

#### ‘Good citizens and bad neighbours’

Senescent cells have been described as ‘good citizens’ as the emergence of a senescent phenotype blocks a damaged cell from becoming cancerous, but ‘bad neighbours’ as their accumulation creates toxic tissue environments [3]. A major question posed by persistence of these cells as a salient feature of all organisms that have a significant tissue regenerative capacity is: ‘Why should the senescent phenotype exist at all?’ Or more specifically: ‘Does the persistence of these cells as tissue components following geroconversion (defined as emergence of senescent phenotype) confer any particular evolutionary advantage to a particular species?’ A speculative analysis of their evolutionary role may be accomplished by considering alternative mechanisms for cancer prevention, which is the primary advantage that senescent cells confer on an organism [3].

#### Evolutionary significance of senescent cellular phenotype (an open question)

The conversion of damaged cells to senescent forms as opposed to cancerous transformation is of obvious advantage, particularly during early life when rates of cell division and tissue/organ growth are high – offering enhanced opportunity for propagation of somatic mutations. During this phase of development, the percentage representation of senescent forms is probably too low to significantly impair tissue function. This developmental phase coincides with peak reproductive proclivity, allowing expansion of a particular population [85]. What then, is the advantage to a species of accumulation of senescent cells? One evolutionary alternative to senescence is activation of an apoptotic response. Apoptotic deletion of lightly or moderately damaged cells which is the stress intensity level favouring senescence [47] would almost certainly have evolved if doing so were to the advantage of a species – defined as size of a population of individuals surviving through reproductive years. Indeed, as senescent cells are deleterious to an organism, would not the disappearance of this trait be a species benefit?

Certainly, reduced tissue representation of senescent cellular phenotypes would be advantageous to a particular individual. Accumulation of these cells creates tissue environments that promote dysregulated inflammation and cancer [3] and exhausts regenerative capacity of progenitor stem cells [44]. Based on the comprehensive picture of the biological role of senescent cells, it seems likely that an evolutionary trend resulting in deletion of incipient cancer cells by ‘gatekeeper’ tumour suppressor functions such as p53/p21 and pRB/p16 [3] would be sufficient to safeguard a species against pathological propagation of somatic mutations – with cancer as a major outcome. Again, the question arises: ‘What advantage does persistence of the senescent cellular phenotype have for a particular species?’

One possible answer is that the major evolutionary role of senescent cells is to limit the lifespan of a particular organism within a species. Their absence might contribute to longer individual lifespans, as a major effect of these cells is to limit the regenerative capacity of progenitor populations [44]. Whether depletion of senescent forms in a particular organism would allow increased lifespan is highly speculative. However, at the time of this writing, insight into how development of senescent phenotypes may be blocked and even reversed is promising and will be discussed in the next section.

The evolution of a longevistat, whether cellular senescence or some other trait, would only occur if there is a general selective advantage for it. Some arguments can be made that limits on lifespan for members of a particular species would favour expansion of its size over time. But equally valid arguments can be made that survival of a core group of very long-lived individuals would be enormously beneficial for a species. This is certainly true for human beings. These arguments are speculative and will inevitably become highly political if age-intervention technology becomes available.

If indeed, senescent cell forms limit the lifespan of an individual, then this cellular phenotype constitutes a true ‘longevistat’ – and therefore becomes a priority target for age-intervention research. The question of whether senescent cellular phenotypes have evolved to limit individual lifespan for the benefit of a species will undoubtedly remain a subject of debate for a long time. It is nevertheless clear that persistence of these cells is detrimental to an individual once their major protective function, cancer prevention, has been accomplished. Accordingly, a major research direction will include strategies for eliminating senescent cells as they form and replacing them with healthy tissue.

### Contribution of toxic aggregates to proteinopathies, progeroid syndrome and ageing

Insight into how senescent cellular phenotypes form and strategies for suppressing their development and pathological features have come about as a result of investigations into the molecular mechanisms of progeroid diseases, which are disorders that resemble accelerated ageing. As described in Section I-5 of the present review, a progeroid syndrome of particular interest in oxidative stress-mediated manipulation of age-associated tissue epigenomic profiles is HGPS. Its major symptoms resemble physical manifestations of ageing, including wizened appearance/wrinkling and other age-related physical deterioration. Death typically occurs as a result of heart failure or stroke, with accompanying atherosclerosis and other cardiovascular disorders at about age 13 [86]. Cells from HGPS-afflicted individuals express high levels of progerin, a mutant form of the nuclear envelope-associated intermediate filament prolamin A, which also accumulates in normal, non-HGPS persons with advancing age. Progerin activates signalling pathways (including DDR kinases) that profoundly disrupt cell structure and homoeostasis, causing shortened telomeres and rapid progression to cell cycle arrest followed by hyperactivation of mTOR and geroconversion into senescent forms [87].

#### mTOR inhibition promotes autophagy, and suppresses cellular senescence

Presence of cellular debris and damaged organelles stimulates autophagic activity at rates sufficient under some conditions to restore cell viability. But oxidative damage, or particularly deleterious mutant proteins such as progerin promotes DDR activity at high levels, causes hyperactivation of mTOR, which suppresses autophagy, allowing damage and debris to stimulate emergence of the senescent phenotype [27, 87]. Remarkably, the drug rapamycin (sirolimus) inhibits mTOR activity to levels allowing for reconstitution of a cell's autophagic capacity, clearance of progerin and other debris and prevention or reversal of geroconversion in cells from HGPS patients [44, 88], and also in non-HGPS mammalian cells [89]. mTOR is thus a major contributor to cellular senescence and to whole-organism ageing due to its geroconvertive ability; moreover, inhibition of mTOR allows enhanced autophagic degradation of toxic aggregates accumulating in the cell [90, 91].

#### Autophagic contribution to resolution of neurodegenerative disease

Intracytoplasmic accumulation of toxic aggregates are the primary cause of several major neurodegenerative syndromes, including tauopathies, Parkinson's, Huntington's chorea, Alzheimer's disease and other ‘proteinopathies’ in which intracellular oxidative stress and subsequent tissue inflammation results from disruption of normal cellular homoeostasis by toxic aggregates [92]. Thus, therapies which stimulate autophagy through mTOR inhibition are potentially of widespread use in therapy for serious chronic diseases [28].

#### Value of autophagy in suppression of cellular and whole-organism senescence

The aforementioned effects clearly establish autophagy and drugs, such as rapamycin which promote it, as valuable tools for preventing accumulation of senescent cells – in contrast to the role of autophagy in some settings as a cell death mechanism [27]. The inhibitory effect of rapamycin on ageing of mammalian cells *in vitro*, through augmentation of autophagy, has proven valid in other models, both cell culture and whole organism, and has been shown to extend lifespan in yeast [93], *Caenorhabditis elegans* [94], Drosophila [95] and mice [88]. Moreover, both animal model and human clinical studies show that the drug inhibits atherosclerosis, which is a primary symptom and major cause of death among both progeric and normal elderly human beings [96–98].

#### Significance of progerin to studies of normal ageing

A major challenge posed by use of progeroid models to design strategies for countermeasures against normal ageing is defining the role of progerin. All investigations of this molecule show it to be a major mediator for emergence of the kinds of senescent cellular phenotypes observed in both HGPS and normal mammalian cells [27]. It is, therefore tempting to predict that blocking development of this mutant lamin may prove to be a significant tool in age intervention. Nevertheless, the processes by which progerin contributes to cellular senescence and accelerated whole-organism ageing, may not be a unique characteristic of this molecule, but a general feature of many kinds cellular debris capable of disrupting structure and function of cells.

### Hypothetical model of age-associated physical decline and proposed countermeasures

The foregoing analysis suggests that the fundamental cellular basis for age-associated physical decline may closely resemble proteinopathies underlying neurodegenerative disorders [92] and that treatments that retard accumulation of such debris (such as augmenting autophagy through suppression of mTOR) represent potentially productive age-intervention strategies [27, 85].

#### Aggregate accumulation model of ageing

The concept of age-associated physical decline occurring as a result of a progressive accumulation of cellular damage is not new. The novel features of research into the ageing process explored in the present review are considerations of how senescent cellular phenotypes emerge and adversely affect homoeostasis as these cells become represented at increasingly higher percentages in tissue. According to the proposed model, physical ageing occurs as a consequence of five fundamental processes:

- *Cellular damage*. Typically, this damage takes the form of extracellular trauma resulting in increased levels of oxidative compounds inside cells. It may also occur due to telomere deterioration or activation of the ras oncogene, which internally increase oxidative stress and damage to cellular components.
- *Activation of damage response programs*. Oxidative and other damages activate programs that include major anticancer safeguards through p53- or pRB1/p16-mediated cell cycle arrest. These programs nevertheless promote hypersecretory processes such as mTOR, resulting in further toxic aggregate accumulation and emergence of senescent cells.
- *Senescent cell accumulation*. Senescent cells degrade surrounding tissue and decrease whole-organism viability. Moreover, the selective elimination of these cells in a progeroid mouse model delays the onset of age-related pathologies and reduces the severity of established diseases [23].
- *Increased risk of cancer and inflammatory disease*. A major and progressively increasing effect of senescent cell accumulation is that expression of pro-inflammatory humoural mediators by these cells and their direct interaction with surrounding tissue dysregulates inflammatory processes and also promotes carcinogenic transformation [3].
- *Decreased stem-cell effectiveness and depletion*. Senescent cells inhibit the capacity of stem cells to regenerate tissue function and decrease tissue representation of both stem and healthy quiescent cells [44].

#### Obligate requirements for age intervention

Any medical treatment capable of affecting age-related physical decline must alter tissue cellular profiles as defined in criteria i – iv below.

#### Tissue cellular composition profile criteria

Effective age-intervention strategies must result in a stable alteration in tissue composition of functional cell categories described in Section I-2 of the present review that meet the following criteria: (i) Elevated representation of stem and healthy quiescent cells (Categories I and II respectively); (ii) No representation of cancer cells (Category III); (iii) Minimal-to-negligible representation of senescent cells (Category IV); and (iv) Representation of cells executing death programs (Category V) in ratios that maintain the stability of the aforementioned optimal cellular phenotypic profiles.

#### Role of telocytes in development of age-intervention strategies

The present review has considered how skewing the representation of various cellular phenotypes within a tissue in favour of healthy quiescent or progenitor stem-cell populations may allow sustained tissue function for long, perhaps open-ended, time periods. The foregoing analysis has nevertheless not until this point taken into consideration the influence of telocytes (TC), which are cell species that only recently have been recognized as potentially critical to enabling prolonged maintenance of tissue regenerative capacity and normal homoeostasis [99]. These cellular phenotypes were discovered and named by Professor Lauren iu M. Popescu's research group at Carol Davila University in Bucharest, Romania [99]. They were shown to exhibit ultrastructure and immunophenotype characteristics distinct from other components of the interstitium (stroma), such as polymorphonuclear leucocytes, mast and mesenchymal cells and fibroblasts [99–101]. Telocyte morphology typically includes a very small cell body containing the nucleus and a small volume of cytoplasm, from which several tubular projections called telopodes extend up to several hundred μm from the body, interfacing with immune, vascular, muscle and neural tissue in a three-dimensional network which is increasingly demonstrated to maintain local homoeostasis of a particular tissue through coordination of intracellular communication [99–101]. Ongoing investigations are revealing increasingly important roles for TC in the stabilization of healthy tissue function so as to minimize inflammatory tissue damage to particular organs. For example, Zhao *et al*. reported inverse correlation between the magnitude of infracted zones of rat myocardium and cardiac telocyte density [102]. Telocytes, therefore, function in tandem with stem cells in anatomical locations designated ‘stem-cell niches’, resulting in an enhancement of their engraftment capability [103].

Isolation of TC and comprehensive structural and functional characterization of these cells is made challenging by their diffuse and intimate association with resident tissue and stroma. Nevertheless, development of protocols for augmenting engraftment of progenitor cells by manipulation of telocyte activity is a strategy which may become critical for prolongation of healthy tissue homoeostasis. One strategy for accomplishing this objective without requiring purified preparation of TC are dose–response experiments in which electron microscopy, immunohistochemistry, immunofluorescence, time-lapse videomicroscopy and whole-cell patch voltage-clamp-based assays were used to describe ultrastructural, immunohistochemical and electrophysiological characteristics in human myometrium [104]. Related investigations undertaken to assess drug effects on telocyte function often include dose-response studies in which agents such as sildanafil, known to enhance stem cell engraftment [105] are evaluated for correlations between drug dosage, progenitor cell stability within a tissue and telocyte functional and phenotypic characteristics as described by Cretoiu *et al*. [104]. Such experiments would be best structured to use tissue models in which TC have been previously studied, combined with drug treatments known to improve progenitor stem-cell engraftment stability. Using this general approach, insight will be gained that allow drug combinations to be optimally configured to affect telocyte function in ways expected to contribute to tissue conditioning in development of age-intervention protocols.

The foregoing findings and experimental strategies underscore the role of TC as a kind of MSC. This capacity is particularly relevant to development of strategies in which commercially available MSCs such as PLX-I are used as an approach to stabilize engrafted progenitor cells into a wide range of tissue, concurrent with depletion of senescent forms, with the objective of prolonging regenerative capacity of a particular tissue. Indeed, as both TC and MSC may share a wide range of overlap in tissue function, the combined effect of PLX-I and telocyte activity may prove to be a very powerful approach to stabilization of engrafted progenitor cells in many tissues. This possibility is explored in the following section, which proposes strategies for sustained skewing of cellular profiles of a tissue as an element of life extension.

#### Theoretical methods for achieving stable tissue cellular profiles

The following prediction of a general approach for meeting the above five tissue cellular composition criteria is highly speculative. Moreover, some of the specific methodology suggested in the five methodological features described below is not currently practical. However, each method relies on increasingly sophisticated technology available at the time of this writing – and may become practical based on rapidly evolving understanding of the relationship between the activity of cells in tissue and whole-organism physical status. Currently, an optimal age-intervention treatment will likely include the following seven major features:

- *Establishment of a stable niche in each tissue for enhancement of progenitor stem-cell engraftment*. Creation of such niches requires that stromal matrices for each tissue be enhanced with respect to their capacities to allow stable engraftment of immigrant progenitor cells. The cellular makeup of stroma varies from tissue to tissue. For example, polymorphonuclear leucocytes Pleuristem PLX-I cells currently offer the best commercially available choice for ready availability of precursors to these cells from umbilical cord blood and ease of *in vitro* expansion, along with their non-immunogenicity and immunoregulatory properties [39]. Three technical challenges must be overcome before a PLX-like product may be used to simultaneously alter the stromal environment of multiple tissues as part of a life extension protocol. First, before PLX, similar products may be used in this manner; strategies must be developed to improve targeting of selected PLX (or other MSC) variants into selected tissues. Precedents have been set by Pleuristem and affiliated clinicians in PLX-1-augmented restoration of haematopoietic capability of bone marrow for BMT patients [39]; however, to use a family of PLX products as a general mediator of tissue regeneration for all tissues in an organism, an integrated array of PLX products and protocols must be developed. Development of these protocols should assess the impact of PLX infusion on telocyte density within a particular tissue, as described by Zhao *et al*. [102] and where possible, use outcome to optimize PLX treatment parameters.
- *Periodic replenishment of oligopotent progenitor stem cells*. An essential requirement for age-intervention methodologies based on directed skewing of cellular tissue profiles is a stem cell capable of migrating to a variety of tissues in the body and undergoing differentiation into progenitor forms for each tissue. Where possible, autologous progenitor cells or multi-potent allogeneic cells capable of migrating into selected target tissues may be administered. Second, protocols must be developed for vectoring progenitors of specific tissues efficiently into those tissues.
- *Cancer-suppressive components*. This safety feature is essential, based on the use of novel stem-cell lines as the basis for replacement of progenitor stem cells. Alterations in the proliferative potential of the stem-cell line from which the therapeutic stem cells were derived may significantly enhance the risk of cancer. One approach to the elimination of tumours arising from the therapeutic line is to create it to be transgenic for a gene product capable of inducing a cell death program, with apoptosis being the optimal result due to the benign interaction between its products and surrounding tissue (as opposed to necrosis which may be pro-inflammatory). The transgenic manipulation needed to accomplish this is carried out routinely at the time of this writing, with an example provided by the work of researchers at Dana Farber who created mice with genomes containing expression cassettes for the telomerase, inducible by the drug 4-OHT [14]. In a similar manner, a drug-responsive element capable of expressing a pro-apoptotic gene product would be inserted into the age-intervention stem cell. If this were combined with insertion of a constitutively expressed biomarker ‘tag’ element similar (for example) to prostate-specific antigen (PSA), a person could be monitored for precipitous increases in the biomarker and treated with the inducer if such increases occurred. This basic strategy would necessarily require elaboration in the form of backup inducers of cell death programs, alternate drug-inducible expression cassettes and other refinements that would potentially create a safe, effective and versatile stem-cell line capable of providing ongoing tissue regenerative capacity, with minimal risk of stem cell-derived cancer.
- *Enhancement of autophagy at levels sufficient to retard geroconversion of (all) cells*. If a major underlying cause of cellular senescence is accumulation of toxic aggregates, then strategies that augment autophagy will contribute to the overall effect of the age-intervention therapy on tissue. Here, it is prudent not to rely on a single approach to enhance clearance of toxic cellular aggregates. For example, although rapamycin may suppress geroconversion through mTOR inhibition and a resulting amplification of autophagy [27], administering high doses of this drug in conjunction with other elements of the age-intervention program described above is unlikely to be beneficial and would likely be toxic to the patient. Optimal clinical implementation of the proposed program might include subtoxic doses of rapamycin, along with other inducers of autophagy such as sirtuin enzymes. Sirtuins may be induced by dietary caloric restriction and the grape polyphenol resveratrol [106]. Their increased activity suppresses mTOR and up-regulates autophagy [27, 85]. Hence, several relatively benign treatments may be given to a recipient along with low-dose rapamycin and the aforementioned stem-cell therapy to maintain and enhance tissue viability.
- *Modulation of oxidative stress levels to achieve desired balance of cell death program activation: Use of oxidative stress as a cell death ‘rheostat’*. As previously described, if the intensity of oxidative stress to which a particular tissue is subjected is altered, the cellular makeup of the tissue also changes. For example, increased oxidative stress levels influence elevated rates of necrotic, apoptotic and senescent cells [47]. This phenomenon theoretically provides a way to ‘sculpt’ tissue composition by varying oxidative stress on a tissue in ways that produce death responses towards desired ratios of cells in apoptosis, necrosis, necroptosis or autophagy, depending on the intended tissue representation of various cell types. A typical example of this strategy might include the use of a drug such as 6-hydroxydopamine which increases oxidative stress on tissue [49], administered in conjunction with sour cherry seed biflavones, a treatment that has been shown to powerfully inhibit oxidative stress [107]. Herein are the elements of a ‘rheostat’ for adjusting the representation of each cell death program in a particular tissue. Ongoing *in vitro*, animal and human trials are expected to reveal the dosage combinations that will produce desired ratios of each death program, allowing increasingly precise tissue engineering.
- *Depletion of senescent cell phenotypes*. The augmentation of tissue with potentially rejuvenating populations of stem cells will be only minimally effective if progressively increased numbers of senescent cells counteract their beneficial effects. This constraint on stem cell-based age-intervention therapies is underscored by studies at the Mayo Clinic College of Medicine showing profound attenuation of age-related physical decline in progeroid mice due to elimination of senescent cells [23]. For this reason, it is desirable to deplete tissue of senescent cell phenotypes wherever possible. Success of the aforementioned investigation was made possible by designing a transgenic mouse strain in which a critical signal to initiate the development of cellular senescence activated a death program in the presence of a drug inducer. Such a targeting strategy is obviously not applicable to a clinical setting. The objective of the selective ablation of senescent cells, therefore presents some of the same challenges as selective targeting of tumours. Similar to cancer, senescent cells are derived from host tissue and thus not subject to immune deletion – although some of their features may be useful in developing immunotherapeutic countermeasures against them. Fortunately, unlike malignancies, senescent cells are tolerated by the host for long periods of time, exerting their detrimental effects in a dose-responsive manner manifested as the major symptoms of ageing.
- *Limitations on characterization of cellular phenotypic profiles in ageing tissue*. Ongoing characterizations of the senescent cellular phenotype are likely to yield progressively improving targeting strategies against these cells – that are likely to become major components of age-intervention programs. Such research has nevertheless been constrained by the lack of an animal model in which phenotypic representation of cells in tissues of an individual become skewed in ways capable of counteracting age-related physical decline. Recently, however, work by several investigators, notably Kubota *et al*. at the Seto Marine Biological Laboratory at Kyoto University, have demonstrated remarkable properties of hydrozoans and related marine organisms that are proving to be an increasingly important tool for dissecting the age-related cellular kinetics of a potential use in design of tissue regeneration strategies in vertebrates [108]. A summary of this evolving research is provided below:

#### Reversible ontological progression in cnidaria: a unique animal model for sustained tissue regeneration

Approaches designed to significantly mitigate age-related physical deterioration are likely, within limits, to mimic an adaptive strategy utilized by several species within the phylum Cnidaria, which include free-swimming jellyfish and sessile polyps. Some of these animals have evolved a unique regenerative process that enables an individual to undergo a complete reversal of ontological development in response to potentially life-threatening environmental stressors and also to sexual maturity, which marks the onset of whole-organism senescence [109]. The ability to regress into an immature form from which the adult may regenerate, allows the animal to evade death resulting from some kinds of external threats and also from age-related deterioration following sexual maturity. One interesting theoretical consequence of this phenomenon is that individual animals able to avoid death by predation, accident or disease might live for very long time periods. At the time of this writing, the question of whether median life spans for these species are truly open-ended has not been definitively answered, but has become the subject of increasingly intense investigation.

Particular interest is currently focused on correlation between a hydrozoans's longevity potential and circadian timing of spawning activity [109]. Spawning behavior exhibited by many Cnidarian taxa, includes stages in which sexually mature adults spawn larvae that may affix to a stationary object to form colonies of polyps. Immature adult (medusa) forms are released from the surface of polyps as buds which grow into mature (medusa) forms, which when sexually mature, mate and perpetuate the cycle [109]. The RD program is activated in a medusal form (either immature or adult) by external stress or in adults by senescence-associated physiological deterioration following sexual maturity. The RD process in adults is very dramatic and includes shrinkage of tentacles to bud-like appendages, redistribution of major anatomical features and contraction of the entire organism into a ball-like stage, which may the affix to a stationary object, forming appendages called stolons from which a new polyp colony may emerge [109].

The cellular signalling mechanisms underlying RD remain poorly defined. It is known that the process is dependent on a highly specialized form of metaplasia, which is a stress-response pathway in which the cellular composition of a tissue changes to meet novel environmental challenges [110]. Metaplasia is a process widely distributed in the animal kingdom. A well-known example in higher vertebrates is the reaction to cigarette smoke of stem cells resident in airway connective tissue, which are stimulated to differentiate into stratified squamous epithelium, which replaces ciliated pseudostratified columnar respiratory epithelial cells [111]. Such changes in tissue function arise as a result of stress-related signalling cues acting to terminally differentiate progenitor stem cells for a particular kind of tissue.

Tissues of sexually mature, RD-capable Cnidarians contain cells which respond to environmental and internal cues by regression into less terminally differentiated sexually immature forms, which collectively develop into polyps, or immature form of the animal which is physically ‘young’ and may regenerate the adult form [110]. These remarkable organisms are the only known examples of metazoans capable of potentially extending longevity by a cellular process termed ‘transdifferentiation’ that results in complete reprogramming of diploid cell differentiation into a primitive form resembling embryonic or foetal tissue, capable of developing into an adult – in essence living a new life. Regeneration of RD-capable adults from polyps may not, however, precisely represent sustained long life of a particular individual. With the exception of a few founder cells from which the new polyp develops, the adult does not survive as a distinct individual. Tissue turnover is essentially total.

#### Jellyfish, stem cells and human beings

Analysis of the human ageing process using a cnidarian model would for the purpose of most parameters be considered ‘ambitious’. Nevertheless, the age-intervention strategy proposed above in Section III-3 makes use of the most important underlying feature of the *Turritopsis nutricula* survival paradigm. Individual *T. nutricula* animals maintain their diploid genetic identity by periodically regenerating the entire organism from specialized cells that have transdifferentiated into an early juvenile state. At its most fundamental level, this is exactly the course of action proposed in Section III-3 as a hypothetical ageing countermeasure. If autologous stem cells are used as the basis for such a countermeasure, some degree of transdifferentiation into a more primitive form will be necessary. If at some point the technical challenges to apply proposed process are met, regeneration might be accomplished with retention of the physical integrity of an individual. Senescent cells would be ablated (ideally) and healthy tissue (including progenitor stem cells) would be continually replenished by infusion of the yet-to-be-created ‘Trinity’ cell. The term ‘Trinity’ as used in the present context, refers to the functional characteristics of a synthetic cellular phenotype genetically altered to respond to drugs or varying levels of oxidative stress by activation of signaling processes that lead to death or survival *via* three major outcomes, defined by apoptosis, necrosis or autophagy – with intermediate pathways such as necropoptosis [31, 33] also represented as potential outcomes. The proposed ageing countermeasure strategy is speculative. However, with certain refinements, this basic plan may be workable, based on its reliance on technology which is either currently available or likely to emerge in the foreseeable future.

## Conclusions

Recent advances in stem-cell technology, together with a better understanding of mechanisms by which oxidative stress activates the cell death programs, apoptosis in particular, has allowed for the development of a framework for defeating the normal ageing process that may prove workable. A specific clinical method for counteracting an age-related physical decline must alter the cellular composition of an organism's tissues in ways that meet three major criteria: First, an organism's pool of progenitor stem cells must be continually replenished. Second, replacement stem cells must be capable of migration to, and establishment in each of the body's tissues in a configuration that minimizes the risk of stem cell-associated cancer. Third, the emergence of senescent cells and their activity must be suppressed. As described in Section II of the present review, the evolving understanding of known mechanisms leading from oxidative stress to activation of particular cell death programs has allowed development of strategies by which externally applied stressors may reproducibly skew the death/survival programs execution by cells within a tissue towards apoptosis, necrosis, necroptosis or autophagy. The result has been the development of a broad strategy, which may be termed ‘necroapoptophagy’, in which tissue may be ‘sculpted’ by the differential induction of each of the four aforementioned programs leading to cell death (or restoration of healthy cellular function as a potential outcome of autophagy). As laboratory and eventual clinical use of necroapoptophagy becomes increasingly sophisticated and precise in achieving the desired tissue cellular profiles, clinically useful strategies for counteracting normally occurring physical ageing may emerge.
